# Association of Body Mass Index with All-Cause and Cardiovascular Disease Mortality in the Elderly

**DOI:** 10.1371/journal.pone.0102589

**Published:** 2014-07-11

**Authors:** Chen-Yi Wu, Yi-Chang Chou, Nicole Huang, Yiing-Jenq Chou, Hsiao-Yun Hu, Chung-Pin Li

**Affiliations:** 1 Institute of Public Health, National Yang Ming University, Taipei, Taiwan; 2 Department of Dermatology, Taipei City Hospital, Heping Fuyou Branch, Taipei, Taiwan; 3 Department of Education and Research, Taipei City Hospital, Taipei, Taiwan; 4 Institute of Hospital and Health Care Administration, National Yang-Ming University, Taipei, Taiwan; 5 Division of Gastroenterology, Department of Medicine, Taipei Veterans General Hospital, Taipei, Taiwan; 6 National Yang-Ming University School of Medicine, Taipei, Taiwan; Boston University, United States of America

## Abstract

**Objectives:**

To evaluate the associations of body mass index (BMI) with all-cause, cardiovascular disease (CVD), and expanded CVD mortality in the elderly.

**Design:**

Observational cohort study.

**Setting:**

Annual physical examination program for the elderly from 2006 to 2010.

**Participants:**

We included 77,541 Taipei residents aged ≥65 years (39,365 men and 38,176 women).

**Measurements:**

BMI was categorized as underweight (BMI<18.5), normal weight (18.5≤BMI<25), overweight (25≤BMI<30), grade 1 obesity (30≤BMI<35), or grade 2–3 obesity (BMI≥35). Mortality was ascertained by national death files.

**Results:**

Underweight (hazard ratios [HRs] of all-cause, CVD, and expanded CVD mortality: 1.92, 1.74, and 1.77, respectively), grade 2–3 obesity (HRs: 1.59, 2.36, and 2.22, respectively), older age, male sex, smoking, and high fasting blood sugar were significant predictors of mortality. Meanwhile, being married/cohabitating, higher education, alcohol consumption, more regular exercise, and high total cholesterol were inversely associated with mortality. Multivariate stratified subgroup analyses verified smokers (HRs of all-cause, CVD, and expanded CVD mortality: 3.25, 10.71, and 7.86, respectively, for grade 2–3 obesity), the high triglyceride group (HRs: 5.82, 10.99, and 14.22, respectively for underweight), and patients with 3–4 factors related to metabolic syndrome (HRs: 4.86, 12.72, and 11.42, respectively, for underweight) were associated with mortality.

**Conclusion:**

The associations of BMI with all-cause, CVD, expanded CVD mortality in the elderly are represented by U-shaped curves, suggesting unilateral promotions or interventions in weight reduction in the elderly may be inappropriate. Heterogeneous effects of grades 1 and 2–3 obesity on mortality were observed and should be treated as different levels of obesity.

## Introduction

Overweight/obesity has become a serious public health problem accompanying changes in dietary habits and physical activity and is increasing to epidemic proportions in most industrialized counties [1], [2]. Many in-depth studies have investigated the impacts of body weight on mortality. However, the elderly are frequently excluded from such studies, particularly those presenting with compromising comorbidities. Cardiovascular disease (CVD) represents the main cause of mortality in the elderly population; obesity is a significant risk factor for CVD and other CVD-related diseases such as hypertension, type II diabetes mellitus, and dyslipidemia [3],[4]. Nevertheless, clarifying how overweight or obesity influences the survival of the elderly, including all-cause and CVD mortality, may be critical.

Numerous reports in the previous decade document an obesity paradox: overweight or obese individuals are reported to have a mortality risk lower than that of normal weight individuals [5]–[10]. This contradicts the well-accepted empirically based idea that obesity confers increased mortality. This controversy is frequently debated, and whether such findings can be applied to the elderly, which is the fastest growing population segment in many countries, remains inconclusive. Body mass index (BMI) is a reasonably good measure of general adiposity in the elderly [2]. The effects of increasing BMI on mortality are less pronounced in the elderly than young or middle-aged adults [11]. However, another study reports the obesity–mortality relationship strengthens with age after controlling for confounders [12]. In 2006, Janssen et al. performed a meta-analysis examining the association between elevated BMI and all-cause mortality in the elderly; they report overweight is not associated with a significantly increased risk of mortality, while moderate obesity is associated with a modest increase in risk [13]. In contrast, in 2013, Flegal et al. performed another meta-analysis evaluating the associations of overweight and obesity with all-cause mortality in the elderly; they report overweight is associated with significantly lower mortality and obesity is not associated with higher mortality [14]. Meanwhile, Lin et al. report higher BMI and waist circumference are associated with lower mortality in Chinese long-term care facility residents [15]. These contradictory findings may be attributable to the different follow-up periods and covariates analyzed [16]. Thus, the optimal BMI and effects of being underweight or overweight on the risk of mortality of the elderly remain controversial. Therefore, further evidence is needed to clarify this issue.

This study evaluated the associations of BMI with all-cause mortality, CVD mortality, and expanded CVD in a large cohort of elderly Taiwanese people, who were medically screened in a standardized process and followed-up for 5 years, yielding 254,211 person–years of observation. Expanded CVD was coined as a new category to capture additional deaths traditionally not classified as CVDs, such as diabetes and kidney diseases.

## Methods

### Study population

The main data were from the Taipei Geriatric Health Examination Database. The study cohort comprised 77,541 participants aged 65 years or older, including 39,365 men and 38,176 women, in a standard annular physical examination program for elderly people run by the Taipei City Government from 2006–2010. Identical screening procedures and protocol were used in all qualified hospitals that contracted with the Department of Health, Taipei City Government [17]. The results were centrally managed and stored. Subjects participated in the physical examination program voluntarily and were encouraged to visit on an annual basis. However, only the results from the initial visit were analyzed. Demographic and lifestyle information (e.g., marital status, education level, smoking history, alcohol consumption, and exercising habit) were collected through a self-administered questionnaire. During the medical check-up, blood pressure was measured. Overnight fasting blood was collected for the measurement of serum blood sugar, triglyceride (TG), total cholesterol (TC), glutamic oxaloacetic transaminase (GOT), glutamic pyruvic transaminase (GPT), albumin, blood urine nitrogen (BUN), creatinine, and hemoglobin. Data related to individual identification were removed before all data were released to the researchers. The acquisition and processing of the data were approved by the Institutional Review Board of Taipei City Hospital (approval number: TCHIRB-1020417-E).

### Definition of BMI

Height and weight were measured during examinations using standardized procedures. BMI was calculated as weight in kilograms divided by height in meters squared. This study employed the BMI-based categories defined by the World Health Organization (WHO) of underweight (BMI<18.5), normal weight (18.5≤BMI<25, reference category), overweight (25≤BMI<30), grade 1 obesity (30≤BMI<35), and grade 2–3 obesity (BMI≥35).

### Controlled variables

Baseline data were collected, including age (i.e., 65–69, 70–74, 75–79, 80–84, or ≥85 years), sex, marital status (i.e., single, married/cohabiting), education level (i.e., none, 1–6 years, 7–12 years, or >12 years), smoking (i.e., frequently/occasionally or no), alcohol consumption (i.e., frequently/occasionally or no), and regular exercise (i.e., none, 1–2 times/week, or 3–5 times/week), high fasting blood sugar (FBS) (i.e., no or ≥126 mg/dL), high systolic blood pressure (SBP) (i.e., no or ≥140 mmHg), high TG (i.e., no or ≥200 mg/dL), and high TC (i.e., no or ≥200 mg/dL).

### Outcome variables

The vital status of the 77,541 study subjects as of December 31, 2010 was ascertained by matching cohort IDs with computerized national death files. Information on the causes of death was coded according to the International Classification of Diseases, Ninth Revision (ICD-9) 2006 or ICD-10 2009 and 2010, including all causes (ICD-9: 001–998; ICD-10: A00-Z99), CVDs (ICD-9: 390–459; ICD-10: I00–I99), and expanded CVD (CVD plus diabetes, ICD-9: 250; ICD-10: E10–E14, plus kidney diseases, ICD-9: 580–589; ICD-10: N00–N29).

### Statistical analyses

The relative risks of all-cause, CVD, and expanded CVD mortality were calculated using Cox proportional hazards models. The proportional hazard assumption was examined by plotting the log-log plots of each category of BMI, and the plot showed that the line for each category was straight and parallel. We also tested whether the log hazard ratio function is constant over time. For 19 of the 21 variables, we accepted the null hypothesis, and for 2 variables, we rejected the null hypothesis, i.e., exercise for 3–5 times/week (*P*<0.001) and BMI of 25–29.9 kg/m^2^ (*P* = 0.03). The log-log plots indicated that the data might be suitable for the Weibull distribution parameters estimation. A sensitivity test of the Weibull model was performed. The times of entry and exit were the date of examination and the end of follow-up (December 31, 2010) or death if earlier, respectively. The reference group comprised subjects with 18.5≤BMI<25, and the hazard ratio (HR) for each BMI category was calculated. Two models were used to avoid over-adjusting for factors in the causal relationship between obesity and mortality. The first model was adjusted for age, sex, marital status, education level, smoking, alcohol consumption, and physical activity; models adjusted for these factors are considered to be adequately adjusted [14]. Additional variables closely related to metabolic syndrome, including high FBS, high SBP, high TG, and high TC, were adjusted for in the second model, which was considered an over-adjusted model. Furthermore, the HRs were calculated with grades 1 and 2–3 obesity combined as a single obesity group. Another sensitivity test was performed to calculate the HR with the reference group set as subjects with 23≤BMI<25.

In the model considered adequately adjusted, HRs were calculated for the following specific subgroups: non-smokers, smokers, men, women, young–old (65–74 years), old–old (≥75 years), normal FBS, high FBS (≥126 mg/dL), normal SBP, high SBP (≥140 mmHg), normal TG, high TG (≥200 mg/dL), normal TC, and high TC (≥200 mg/dL) as well as and 0, 1, 2, or 3–4 risk factors for metabolic syndrome (i.e., high FBS, high SBP, high TG, and high TG).

All analyses were performed using SAS 9.3 (SAS Institute Inc., Cary, NC, USA) and the STATA 10.0 (STATA Corp, College Station, TX, USA). The level of significance was set at *P*<0.05.

## Results

The percentages of individuals in each BMI category, selected characteristics of all individuals, and mortality during follow-up are shown in Table 1. The mean ± SD age of participants was 73.1±6.6 years. The 5-year study period yielded 254,211 person-years of follow-up, with a mean follow-up of 3.3±1.3 years. According to the WHO definition, the BMI of 3.5%, 57.8%, 33.4%, 4.8%, and 0.5% of the elderly population was classified as underweight, normal weight, overweight, grade 1 obesity, and grade 2–3 obesity, respectively. During the 5 years of follow-up, 3,842 (5.0%) deaths occurred, including 877 (22.8% of all deaths) deaths due to CVD and 1,116 (29.0%) deaths due to expanded CVD. Among all participants, 9,923 (12.8%), 30,745 (39.6%), 9,349 (12.1%), and 36,919 (47.6%) had high FBS, SBP, TG, and TC, respectively; 57,166 (73.7%) had at least one of these factors.

**Table 1:** Baseline Characteristics of Participants and Deaths by 5 Categories of BMI Category.

Variables	All	BMI (kg/m^2^)									
		<18.5 Underweight		18.5–24.9 Normal weight		25–29.9 Overweight		30–34.9 Grade 1 obesity		≥35 Grade 2–3 obesity	
		*n*	%	*n*	%	*n*	%	*n*	%	*n*	%
Total	77,541	2,708	3.5	44,842	57.8	25,896	33.4	3,684	4.8	411	0.5
Age (years)											
65–69	28,492	724	2.5	16,527	58.0	9,619	33.8	1,445	5.1	177	0.6
70–74	18,381	524	2.9	10,212	55.6	6,549	35.6	967	5.3	129	0.7
75–79	16,288	633	3,9	9,371	57.5	5,485	33.7	735	4.5	64	0.4
80–84	9,888	466	4.7	5,941	60.1	3,054	30.9	391	4.0	36	0.4
≥85	4,492	361	8.0	2,791	62.1	1,189	26.5	146	3.3	5	0.1
Sex											
Male	39,365	1,361	3.5	22.941	58.3	13,536	34.4	1,408	3.6	119	0.3
Female	38,176	1,347	3.5	21,901	57.4	12,360	32.4	2,276	6.0	292	0.8
Marital status											
Single	20,323	898	4.4	11,759	57.9	6,469	31.8	1,068	5.3	129	0.6
Married/cohabiting	57,218	1,810	3.2	33,083	57.8	19,427	34.0	2,616	4.6	282	0.5
Education (years)											
None	6,859	254	3.7	3,462	50.5	2,517	36.7	555	8.1	71	1.0
1–6	21,492	697	3.2	11,408	53.1	7,848	36.5	1,378	6.4	161	0.8
6–12	27,664	996	3.6	16,587	60.0	8,829	31.9	1,123	4.1	129	0.5
≥12	21,526	761	3.5	13,385	62.2	6,702	31.1	628	2.9	50	0.2
Smoking											
Frequently/occasionally	6,849	348	5.1	4,003	58.5	2,199	32.1	273	4.0	26	0.4
None	70,692	2,360	3.3	40,839	57.8	23,697	33.5	3,411	4.8	385	0.5
Alcohol consumption											
Frequently/occasionally	62,420	383	2.5	8,441	55.8	5,641	37.3	598	4.0	58	0.4
None	15,121	2,325	3.7	36,401	58.3	20,255	32.5	3,086	4.9	353	0.6
Regular exercise											
None	8,487	411	4.8	4,454	52.5	2,901	34.2	620	7.3	101	1.2
1–2 times/week	28,780	1,039	3.6	16,367	56.9	9,717	33.8	1,461	5.1	196	0.7
3–5 times/week	40,274	1,258	3.1	24,021	59.6	13,278	33.0	1,603	4.0	114	0.3
High FBS (≥126 mg/dL)											
Yes	9,923	117	1.2	4,770	48.1	4,043	40.7	868	8.8	125	1.3
No	67,618	2,591	3.8	40,072	59.3	21,853	32.3	2,816	4.2	286	0.4
High SBP (≥140 mmHg)											
Yes	30,745	817	2.7	16,387	53.3	11,429	37.2	1,884	6.1	228	0.7
No	46,796	1,891	4.0	28,455	60.8	14,467	30.9	1,800	3.9	183	0.4
High TG (≥200 mg/dL)											
Yes	9,349	50	0.5	4,345	46.5	4,152	44.4	722	7.7	80	0.9
No	68,192	2,658	3.9	40,497	59.4	21,744	31.9	2,962	4.3	331	0.5
High TC (≥200 mg/dL)											
Yes	36,919	1,153	3.1	21,576	58.4	12,240	33.2	1,740	4.7	210	0.6
No	40,622	1,555	3.8	23,266	57.3	13,656	33.6	1,944	4.8	201	0.5
No risk factors*	20,375	1,047	5.1	12,634	62.0	5,948	29.2	693	3.4	53	0.3
1 factor	33,232	1,217	3.7	19,851	59.7	10,652	32.1	1,355	4.1	157	0.5
2 factors	18,787	416	2.2	10,105	53.8	6,993	37.2	1,141	6.1	132	0.7
3–4 factors	5,147	28	0.5	2,252	43.8	2,303	44.7	495	9.6	69	1.3
All-cause mortality	3,842	341	8.9	2,310	60.1	1,032	26.9	135	3.5	24	0.6
CVD-related mortality	877	72	8.2	527	60.1	242	27.6	28	3.2	8	0.9
Expanded CVD-related mortality	1,116	91	8.2	655	58.7	323	28.9	37	3.3	10	0.9

BMI: body mass index; CVD: cardiovascular diseases; FBS: fasting blood sugar; SBP: systolic blood pressure; TG: triglycerides; TC: total cholesterol.

*Factors included high FBS (i.e., ≥126 mg/dL), SBP (i.e., ≥140 mmHg), TG (≥200 mg/dL), and TC (≥200 mg/dL).

The clinical characteristics of each BMI category are presented in Table 2. The composition factors related to metabolic syndrome, including FBS, SBP, diastolic BP, and TG, were significantly higher with higher BMI (for FBS, *P*<0.001; for SBP, *P*<0.001; for diastolic BP, *P*<0.001; and for TG, *P*<0.001). Other laboratory data including glutamic oxaloacetic transaminase (GOT), glutamic pyruvic transaminase (GPT), albumin, blood urine nitrogen (BUN), creatinine, and hemoglobin also showed an association with BMI as the factors related to metabolic syndrome (for GOT, *P*<0.001; for GPT, *P*<0.001; for albumin, *P*<0.001; for BUN, *P*<0.001; for creatinine, *P*<0.001; and for hemoglobin, *P*<0.001).

**Table 2:** Clinical Characteristics of Participants According to the BMI Category.

Variables	All	BMI (kg/m^2^)				
		<18.5	18.5–24.9	25–29.9	30–34.9	≥35
		Underweight	Normal weight	Overweight	Grade 1 obesity	Grade 2–3 obesity
	Mean ± SD	Mean ± SD	Mean ± SD	Mean ± SD	Mean ± SD	Mean ± SD
Fasting blood sugar (mg/dL)	106.0±27.8	96.7±19.7	103.9±26.8	109.0±28.4	115.4±32.8	122.3±39.9
Systolic blood pressure (mmHg)	135.3±19.7	129.2±20.9	133.8±19.7	137.7±19.0	141.0±19.3	143.9±22.4
Diastolic blood pressure (mmHg)	76.2±11.7	72.3±12.1	75.2±11.5	77.8±11.6	79.2±11.9	81.1±14.0
Triglyceride (mg/dL)	126.8±85.0	81.9±44.5	118.6±82.9	141.8±87.3	152.3±89.7	155.9±78.3
Total cholesterol (mg/dL)	199±36.7	194.8±35.5	199.4±36.3	198.8±37.1	198.9±37.6	202.0±39.8
GOT (U/L)	25.1±14.7	26.6±16.6	24.6±14.2	25.6±14.9	27.4±16.9	29.5±22.3
GPT (U/L)	23.4±19.7	20.0±17.1	21.8±18.7	25.6±20.5	28.9±23.6	30.3±25.8
Albumin (g/dL)	4.3±0.3	4.3±0.3	4.3±0.3	4.4±0.3	4.3±0.3	4.3±0.3
BUN (mg/dL)	17.6±6.4	17.3±6.2	17.4±6.4	17.8±6.3	18.4±8.1	18.5±6.5
Creatinine (mg/dL)	1.0±0.5	1.0±0.5	1.0±0.5	1.0±0.5	1.0±0.6	1.0±0.5
Hemoglobin (g/dL)	13.6±1.4	12.9±1.5	13.5±1.4	13.8±1.4	13.7±1.4	13.6±1.4

BMI: body mass index; GOT: glutamic oxaloacetic transaminase; GPT: glutamic pyruvic transaminase; BUN: blood urine nitrogen.

The HRs of all-cause, CVD, and expanded CVD mortality calculated separately in the first (i.e., adequately adjusted) and second (i.e., over-adjusted) models are shown in Table 3. The predictors of mortality were generally similar between models. Underweight (all-cause mortality, HR: 1.92, 95% confidence interval [CI]: 1.71–2.15; CVD mortality, HR: 1.74, 95% CI: 1.36–2.23; expanded CVD mortality, HR: 1.77, 95% CI: 1.42–2.21) and grade 2–3 obesity (all-cause mortality, HR: 1.59, 95% CI: 1.06–2.38; CVD mortality, HR: 2.36, 95% CI: 1.17–4.76; expanded CVD mortality, HR: 2.22, 95% CI: 1.18–4.15) as well as older age, male sex, smoking, and high FBS were significant predictors of all-cause, CVD, and expanded CVD mortality. On the other hand, being married/cohabitating, higher education, alcohol consumption, more regular exercise, and high TC were inversely associated with mortality. The results of the sensitivity test with the Weibull model remain robust. The hazard ratios of non-CVD mortality (i.e., excluding those with CVD mortality) among each BMI category were also calculated, that is, underweight (HR: 1.97, 95% CI: 1.73–2.24), overweight (HR: 0.81, 95% CI: 0.74–0.88), grade 1 obesity (HR: 0.84, 95% CI: 0.69–1.03), and grade 2–3 obesity (HR: 1.36, 95% CI: 0.83–2.24).

**Table 3:** Cox Proportional Model of Factors Associated with All-cause, CVD, and Expanded CVD Mortality.

Variables	All-cause mortality		CVD mortality		Expanded CVD mortality	
	HR^a^ (95% CI)	HR^b^ (95% CI)	HR^a^ (95% CI)	HR^b^ (95% CI)	HR^a^ (95% CI)	HR^b^ (95% CI)
BMI (18.5–24.9)	1.00	1.00	1.00	1.00	1.00	1.00
15.0–18.4 (underweight)	1.92 (1.71–2.15)	1.95 (1.74–2.19)	1.74 (1.36–2.23)	1.79 (1.39–2.29)	1.77 (1.42–2.21)	1.89 (1.52–2.36)
25–29.9 (overweight)	0.82 (0.76–0.88)	0.79 (0.74–0.85)	0.85 (0.73–0.99)	0.83 (0.71–0.97)	0.90 (0.79–1.03)	0.85 (0.74–0.97)
30–34.9 (grade 1obesity)	0.82 (0.69–0.98)	0.78 (0.65–0.93)	0.75 (0.51–1.09)	0.71 (0.49–1.05)	0.77 (0.55–1.07)	0.69 (0.49–0.96)
≥35 (grade 2–3 obesity)	1.59 (1.06–2.38)	1.48 (0.99–2.21)	2.36 (1.17–4.76)	2.24 (1.11–4.52)	2.22 (1.18–4.15)	1.94 (1.03–3.63)
Age (years) (65–69)	1.00	1.00	1.00	1.00	1.00	1.00
70–74	1.58 (1.39–1.78)	1.55 (1.37–1.76)	1.71 (1.30–2.25)	1.69 (1.28–2.22)	1.82 (1.43–2.31)	1.78 (1.40–2.26)
75–79	2.65 (2.37–2.97)	2.58 (2.30–2.89)	3.34 (2.62–4.31)	3.27 (2.55–4.20)	3.34 (2.69–4.16)	3.23 (2.59–4.03)
80–84	4.33 (3.86–4.86)	4.19 (3.73–4.70)	5.22 (4.05–6.72)	5.07 (3.93–6.53)	5.23 (4.19–6.54)	5.06 (4.05–6.33)
≥85	7.60 (6.74–8.58)	7.33 (6.49–8.28)	10.26 (7.90–13.33)	9.93 (7.64–12.92)	9.18 (7.26–11.59)	8.88 (7.02–11.24)
Sex (Female)	1.00	1.00	1.00	1.00	1.00	1.00
Male	2.08 (1.92–2.24)	1.97 (1.82–2.13)	1.71 (1.46–2.01)	1.68 (1.43–1.98)	1.68 (1.46–1.94)	1.64 (1.42–1.89)
Marital status (single)	1.00	1.00	1.00	1.00	1.00	1.00
Married/cohabiting	0.83 (0.77–0.89)	0.83 (0.78–0.89)	0.82 (0.70–0.94)	0.82 (0.71–0.94)	0.79 (0.69–0.89)	0.79 (0.69–0.90)
Education (years) (None)	1.00	1.00	1.00	1.00	1.00	1.00
1–6	0.84 (0.75–0.93)	0.85 (0.76–0.94)	0.87 (0.70–1.09)	0.88 (0.70–1.10)	0.89 (0.73–1.08)	0.90 (0.74–1.09)
6–12	0.71 (0.64–0.79)	0.72 (0.65–0.80)	0.73 (0.59–0.92)	0.74 (0.59–0.93)	0.69 (0.57–0.84)	0.71 (0.58–0.86)
≥12	0.56 (0.50–0.63)	0.57 (0.51–0.64)	0.63 (0.49–0.80)	0.64 (0.50–0.81)	0.60 (0.48–0.74)	0.61 (0.50–0.76)
Smoking (None)	1.00	1.00	1.00	1.00	1.00	1.00
Frequently/occasionally	1.48 (1.34–1.63)	1.48 (1.35–1.63)	1.30 (1.04–1.61)	1.30 (1.05–1.62)	1.29 (1.06–1.57)	1.30 (1.07–1.57)
Alcohol consumption (None)	1.00	1.00	1.00	1.00	1.00	1.00
Frequently/occasionally	0.72 (0.65–0.78)	0.73 (0.66–0.80)	0.66 (0.54–0.81)	0.67 (0.55–0.82)	0.63 (0.53–0.76)	0.64 (0.54–0.77)
Regular exercise (None)	1.00	1.00	1.00	1.00	1.00	1.00
1–2 times/week	0.68 (0.62–0.75)	0.69 (0.63–0.75)	0.72 (0.59–0.87)	0.72 (0.59–0.87)	0.71 (0.60–0.83)	0.71 (0.60–0.84)
3–5 times/week	0.53 (0.49–0.58)	0.53 (0.49–0.58)	0.59 (0.49–0.72)	0.59 (0.49–0.72)	0.53 (0.45–0.63)	0.54 (0.46–0.64)
High FBS (None)		1.00		1.00		1.00
≥126 mg/dL		1.44 (1.32–1.57)		1.23 (1.02–1.48)		1.80 (1.55–2.09)
High SBP (None)	1.00		1.00		1.00	
≥140 mmHg		1.04 (0.98–1.11)		1.13 (0.99–1.29)		1.11 (0.99–1.25)
High TG (None)		1.00		1.00		1.00
≥200 mg/dL		1.02 (0.92–1.13)		1.05 (0.85–1.30)		1.18 (0.98–1.40)
High TC (None)		1.00		1.00		1.00
≥200 mg/dL		0.77 (0.72–0.83)		0.89 (0.77–1.02)		0.84 (0.75–0.96)

a Model 1: adjusted for age, sex, marital status, education level, smoking, alcohol consumption, and exercise status.

b Model 2: adjusted for all factors in Model 1 plus high FBS, high SBP, high TG, and high TC.

BMI: body mass index; CVD: cardiovascular disease; FBS: fasting blood sugar; SBP: systolic blood pressure; TG: triglycerides; TC: total cholesterol.

A sensitivity test combining grades 1 and 2–3 obesity as a single obese group was performed. Compared to normal body weight, this obese group (i.e., BMI≥30) tended to have lower HRs for overall mortality (all-cause mortality, HR: 0.89, 95% CI: 0.75–1.04; CVD mortality, HR: 0.88, 95% CI: 0.63–1.23; expanded CVD mortality, HR: 0.89, 95% CI: 0.66–01.20). Another sensitivity test set the reference group as 23≤BMI<25, and the results were also similar.

**Figure 1:**
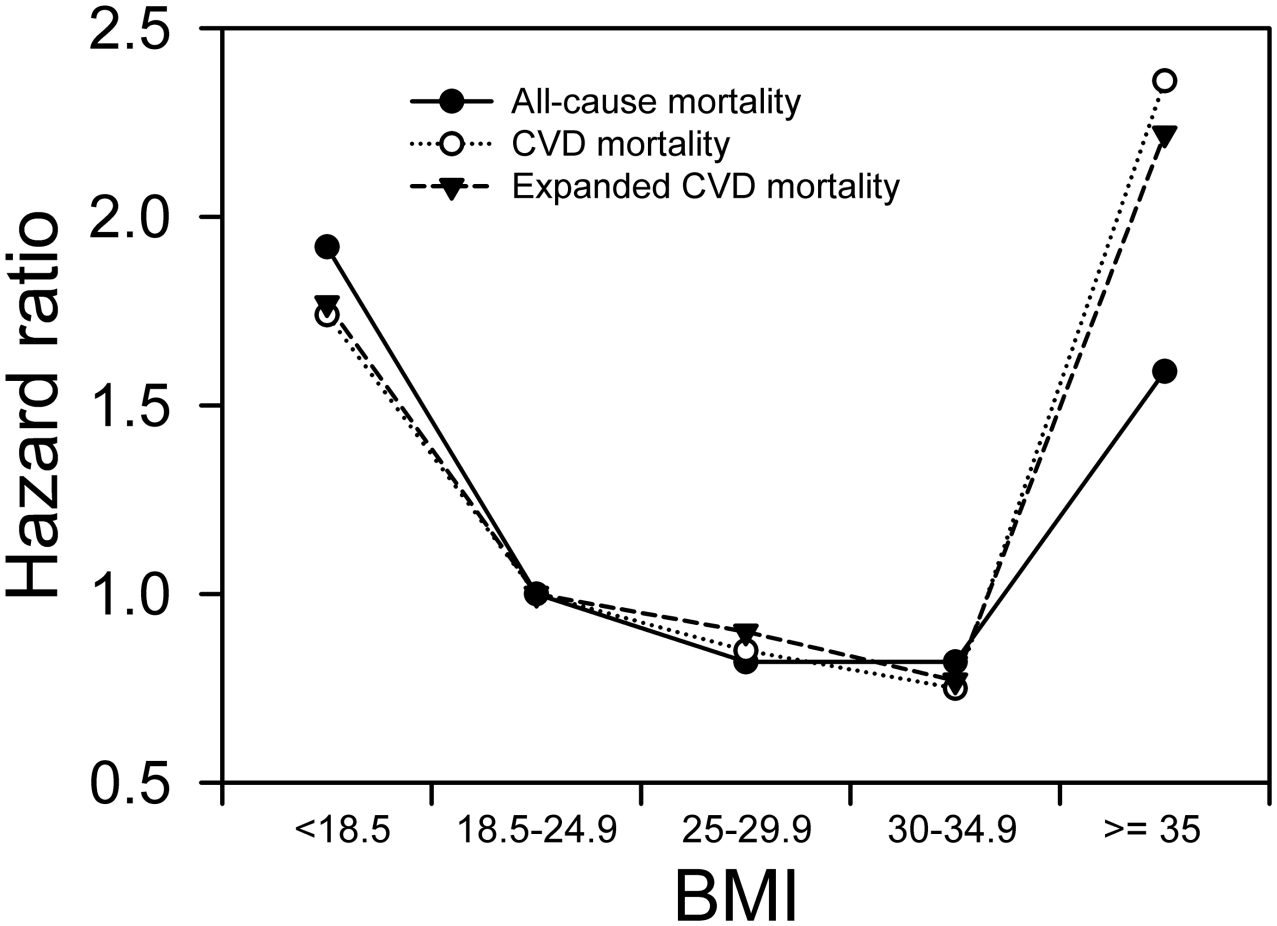
HRs for all-cause, CVD, and expanded CVD mortality according to BMI category.

The associations of BMI with all-cause, CVD, and expanded CVD mortality among the elderly were all represented as generally similar asymmetric U-shaped curves with large flat bottoms and right curves that started to rise significantly at BMI≥35 (Figure 1). The HRs of overweight and grade 1 obesity shared a nadir on the all-cause mortality curve, and the HR of grade 1 obesity was at the nadirs of the CVD and expanded CVD curves. The effects of grade 2–3 obesity were more prominent on CVD and expanded CVD mortality than all-cause mortality.

**Figure 2:**
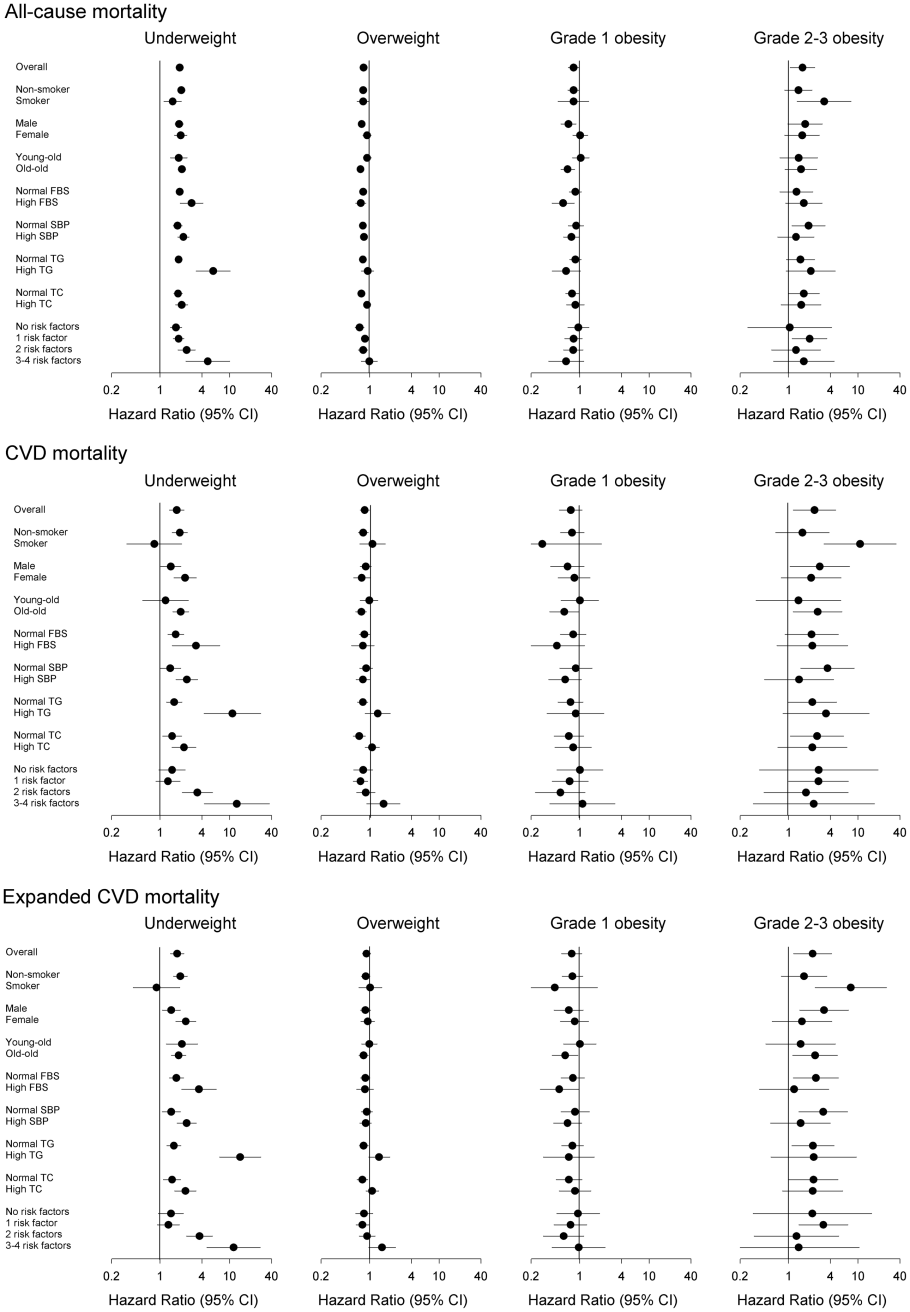
Subgroup analysis of all-cause, CVD, and expanded CVD mortality.

Subgroup analyses are presented in Figure 2. The HRs were almost the same in subgroup analyses. Among smokers, grade 2–3 obesity was more strongly associated with the mortality (all-cause mortality, HR: 3.25, 95% CI: 1.33–7.93; CVD mortality, HR: 10.71, 95% CI: 3.23–35.44; expanded CVD mortality, HR: 7.86, 95% CI: 2.41–25.58). In the high TG group, underweight was strongly associated with mortality (all-cause mortality, HR: 5.82, 95% CI: 3.30–10.25; CVD mortality, HR: 10.99, 95% CI: 4.28–28.22; expanded CVD mortality, HR: 14.22, 95% CI: 7.18–28.17). Among those with 3–4 factors related to metabolic syndrome, underweight was significantly associated with mortality (all-cause mortality, HR: 4.86, 95% CI: 2.36–10.01; CVD mortality, HR: 12.72, 95% CI: 4.31–37.51; expanded CVD mortality, HR: 11.42, 95% CI: 4.73–27.58).

## Discussion

The results of this study show the association between measured BMI and mortality in the elderly. The large sample size enabled subgroup analysis according to particular characteristics. Relative to the normal weight category, the associations of BMI with all-cause, CVD, and expanded CVD mortality in the elderly were generally represented as U-shaped curves with large flat bottoms at overweight and grade 1 obesity, and a right curve that was significantly elevated at a BMI≥35.

The different effects of grades 1 and 2–3 obesity on mortality are noteworthy. When subjects with BMI≥30 were treated as a single obesity group, the overall HR indicated lower mortality; this is because mildly obese subjects outnumbered moderate–severe obese subjects. These results are broadly consistent with those of 3 meta-analyses, which report obesity is associated with lower mortality [13], [14], [18]. However, treating different levels of obesity as a single group might obscure the heterogeneous effects of mild and moderate–severe obesity on mortality.

Some authors suggest lowering the BMI cut-offs for Asian populations [19], while others disagree [20]. Wen et al. report significant mortality risks starting at a BMI≥25 in a much younger Taiwanese cohort [19]. Meanwhile, Gu et al. report underweight and obesity are associated with increased mortality in a Chinese adult population and support the use of a single common recommendation for defining overweight and obesity for all ethnicities [20]. In the present study, the risks of mortality in an elderly Taiwanese population did not differ substantially from those of Western counterparts [13], [14], corroborating the use of a single common recommendation for defining overweight and obesity among all ethnic groups. Furthermore, these findings corroborate the notion that overweight and mild obesity are relatively less harmful in the elderly.

Underweight was associated with increased mortality in previous studies [21]–[23]. In the present study, underweight was associated with all-cause, CVD, and expanded CVD mortality in the elderly. These effects persisted in subgroup analysis. Accordingly, some studies suggest modestly higher body weight may improve survival in some circumstances [5]–[10]. Indeed, overweight and grade 1 obesity were associated with lower mortality risks in the present study, suggesting slightly or mildly elevated BMI increases survival in the elderly. There are some possible explanations for this phenomenon. First, fat storage might be a protective resource in some individuals exposed to acute insults or chronic wasting [7]. Second, BMI cannot distinguish excess body fat from increased lean mass [24]. Third, obesity patients may present with symptoms and morbidities earlier [25], making them more likely to receive prompt medical treatment [26].

Nevertheless, the observed decrease in mortality risk reverses beyond mild obesity. In particular, grade 2–3 obesity was associated with increased risks of all-cause, CVD, and expanded CVD mortality. These results are consistent with those of a previous study, reporting moderate obesity is associated with an increased risk of mortality [13]. Therefore, the present findings may have important implications contrary to the current clinical recommendation that overweight and mildly obese elderly people should lose weight.

In previous studies investigating the association between BMI and mortality, models were considered adequate if adjusted for age, sex, and smoking. Models adjusting for additional factors such as hypertension are considered possibly over-adjusted, because such factors are considered to be a part of the causal relationship between obesity and mortality [14]. Therefore, in order to test the predictive capability of BMI for mortality rather than inferring a causal relationship, evaluating other factors as potential confounders is not a large concern. Regardless, in the present study, the results of the adequately adjusted and over-adjusted models were not substantially different.

Smoking is considered an important risk factor for morbidity and mortality, particularly CVD mortality. Previous studies have analyzed the association between BMI and mortality among non-smokers to avoid the confounding effect of smoking [27]. In the present subgroup analysis, the results of non-smokers were similar to those of the total population. However, it is worth noting the HRs of smokers increased sharply in grade 2–3 obesity, for especially CVD and expanded CVD mortality. The excess mortality risks of BMI among smokers suggest smoking enhances the relationship between higher BMI and survival in the elderly [28].

Diabetes is one of the most significant risk factors for CVD, and BMI is strongly associated with an increased prevalence of CVD independent of metabolic syndrome [29]. In the present study, high FBS was significantly associated with all-cause, CVD, and expanded CVD mortality, whereas high SBP and TG were not. Most interestingly, high TC was negatively associated with mortality, which has been reported previously [30], [31]. In the present study, high TC was associated with higher BMI. Therefore, high TC is possibly associated with better global health conditions among the elderly population. However, the actual role of hypercholesterolemia as a risk factor for mortality among the elderly requires further investigation.

The present study has several limitations. First, this study did not consider preexisting diseases or therapeutic regimens used to control diabetes, hypertension, or dyslipidemia. Baseline laboratory data were used for adjustment in the models and subgroup analysis. Moreover, reverse causation and illness-related weight loss could confound the association between BMI and mortality [32]. A sensitivity test to set the reference group as subjects with a BMI from 23–24.9, which would exclude people who might be underweight due to underlying diseases, would partially reduce the impact of preexisting diseases as the cause of death. Regardless, residual confounding or reverse causation due to preexisting illnesses is reported to have little effect on the estimated relative risks of mortality [8], [33]. Second, the baseline examination data were used to represent all participants in the cohort. These data may have changed over time, leading to complex effects on mortality rates. However, the present results demonstrate the power of a single determination of BMI for predicting mortality risk. Third, the present study analyzed the statistical association between BMI and mortality. Several other factors associated with weight and mortality, such as physical activity level and body composition, could be responsible for part of the observed association. Therefore, mortality data from well-controlled weight loss trials are required to clarify the association between BMI and mortality. Fourth, the voluntary participants of this study may not be representative of the general population. However, because risk comparison was based on internal comparison, the calculated relative risks are a reasonable estimate of those in the general population.

In conclusion, the U-shaped relationship between BMI and mortality in the elderly in the present study suggests unilateral promotions or interventions in weight reduction in the elderly may be inappropriate. Thus, losing weight may not be uniformly beneficial to the health of elderly persons. In particular, it might be necessary to reevaluate strategies related to the pertinence of lowering the weight of those overweight and grade 1 obesity elderly patients.
